# Editorial: Assessing Bipedal Locomotion: Towards Replicable Benchmarks for Robotic and Robot-Assisted Locomotion

**DOI:** 10.3389/fnbot.2019.00086

**Published:** 2019-10-25

**Authors:** Diego Torricelli, Jan Veneman, Jose Gonzalez-Vargas, Katja Mombaur, C. David Remy

**Affiliations:** ^1^Spanish National Research Council (CSIC), Madrid, Spain; ^2^Hocoma AG, Volketswil, Switzerland; ^3^Ottobock SE & KGaA, Duderstadt, Germany; ^4^Optimization, Robotics & BIomechanics (ORB), Institute of Computer Engineering (ZITI), Heidelberg University, Heidelberg, Germany; ^5^Institute of Nonlinear Mechanics, University of Stuttgart, Stuttgart, Germany

**Keywords:** benchmarking, wearable robots, humanoids, walking, balance, prosthetics and exoskeletons

## Introduction

Human-centered bipedal robots, such as exoskeletons, powered prostheses and humanoids are demonstrating increasing levels of functionality, reliability, and safety, and are now breaching the barrier of surviving in a rapidly evolving market. In this crucial process, the lack of accepted standards to evaluate the different facets of robotic performance is hampering the efficient introduction of these new technologies into the different application domains, each of them characterized by the needs, requirements, and regulations of diverse users. A systematic benchmarking methodology to assess robotic systems on a quantitative and reproducible basis is eagerly awaited, as demonstrated by the numerous workshops and discussions on this topic in the most relevant international forums of the last 5–10 years. Benchmarks allow comparing systems to each other and against accepted references, and are therefore useful not only to end-users in search for the most suitable solution, but also to developers aiming to identify and solve the critical shortcomings of their machines. Solid benchmarks usually arise from research results, and are eventually converted into international standards after an iterative process that can last years. It can be argued that the more effort is done by the research community in identifying effective benchmarking methods, the easier it will be to arrive at a complete and relevant set of standards that will improve and accelerate the introduction of new robots in people's lives.

In the field of bipedal walking and posture, a huge body of literature focused on understanding or replicating human performance. Yet, just a few works specifically focused on proposing reproducible metrics that could be potentially converted into benchmarks for human-centered robotic devices. This Research Topic is a first step in this direction. We gathered a total of 16 original research articles from the fields of gait analysis, motor control, simulation, robotics and rehabilitation, covering together most of the aspects of bipedal skills, with a clear focus on identifying measurable metrics of performance. We believe and hope that the works presented in this collection can demonstrate how the process of identifying useful benchmarks of human and human-like robotic motion is possible, and encourage the scientific community to join the efforts in this ambitious goal.

## Assessing, Understanding, and Replicating Bipedal Locomotion

In science, quantitative measures are necessary to understand the basic principles behind behavior and to validate or create scientific hypotheses. In robotics, performance measures are mainly used to achieve—and demonstrate—a certain expected level of performance. With the advent of human-inspired robotics, which aims to integrate biological principles into human-like or human-compatible machines, these two facets become strongly intertwined. Replicating human motion into real-life machines requires a deep understanding of the neuromechanical mechanisms at the basis of human behavior and, at the same time, the achievement of levels of performance comparable with those of the human counterpart. *Assessing* the performance of bipedal systems, being them humans, robots, or the combination of both (as happens in wearable robotics), is therefore a necessary component to bridging the two sides of the same coin: *understanding* and *replicating* human-like locomotion. The articles collected in this Research Topic provide a remarkable example of how these three aspects are strongly enlaced, and how they can be translated into domain-specific metrics.

Three articles of this collection specifically focused on understanding and quantifying human balance, which is a crucial aspect for both walking and standing. Vlutters et al. aim to understand how humans deal with balance perturbations during walking, by experimentally testing reactions after pushes with different amplitudes, direction, and timing. Using simple metrics based on foot placement location, placement timing, and COM velocity, they demonstrate that human response is highly dependent on the timing of the perturbation, and potentially usable as a benchmark of human-like balance reactions. Mergner and Lippi present a comprehensive framework to test the posture control of human-like bipedal systems. The framework, based on a deep knowledge of the sensorimotor mechanisms of human balance, describes the experimental procedures to replicate the most relevant physical disturbances, and the metrics to quantify both performance and human likeness. Liu et al. investigate the causal relationship between spatiotemporal asymmetries and impaired reactive control of balance, by using two main metrics, i.e., whole-body angular momentum and Floquet analysis. These two metrics may be useful in grasping stability measure in impaired and robot-assisted gait.

Five articles focused on metrics and criteria aimed to evaluate or predict the effects of walking with prosthetic devices. Hu et al., based on the fact that bipedal walking requires interlimb coordination, demonstrate that neuromechanical information from both limbs is necessary to improve the prediction of transitions between level walking and stairs or slopes. This work, which has potential benefits for the design of more intuitive and transparent lower limb prostheses, is linked to a datasets of neuromechanical signals from wearable sensors, recorded bilaterally, which has been made available to the community for benchmarking purposes (Hu et al.). Ramasamy et al. propose a novel and subject-specific modeling tool to predict the interaction between a residual limb and a prosthetic device, providing relevant evidence to improve the prediction the potential sites of deep tissue injury generated by the socket-stump interaction forces. Ramakrishnan et al. present a new metric, called combined gait asymmetry metric (CGAM), which provides a unified index of gait asymmetry that combines 11 spatio-temporal, kinematic, and kinetic parameters. This metric has promising potential in the prosthetic field to assess and categorize asymmetries due to different natures, such as leg length or added mass at the leg. Ho Hoang et al. investigated a number of benchmarking criteria to assess the subject-specific strategy of maintaining stability during unimpaired and impaired (i.e., transfemoral amputees) walking. By using optimal control to dynamically reconstruct recorded motions, they showed that human-like stability can be explained by two main indicators, i.e., foot placement and the Residual Orbital Energy.

Five articles proposed metrics and models to estimate and predict the effects of the interaction between humans and lower limb exoskeletons. Mummolo et al. propose an algorithmic framework to evaluate the balance stability of exoskeletons in crutch-less configurations during flat ground walking. They propose new metrics based on center of mass and joint-space dynamics and applied them to the lower-limb exoskeleton Mina-2, demonstrating how this method has interesting potential for the assessment of the future generation of self-balancing exoskeletons. Torricelli et al. proposes a subject-specific musculoskeletal model able to infer the relative motion between the exoskeleton and the human limbs from the kinematic data recorded from the exoskeleton. This approach may contribute to improve predictive control strategies and/or mechanical designs for better human-robot interaction. Gordon et al. applied a set of metrics based on dynamic stability and metabolic cost estimations to exoskeleton-assisted walking under varying conditions (speed, floor inclination, exoskeleton assistance), selecting some of them as potential control variables for adaptive exoskeletons. Gandolla et al. propose an automatic and patient-specific calibration procedure able to detect the best setting control parameters of the lower limb exoskeleton Ekso. The method is based on the maximization of the Gait Metric index, which quantifies the similarity of muscle activations to a reference normative set. Sharbafi et al. propose a control algorithm for exoskeletons based on the virtual pivot point (VPP) concept and simulate its effects on a detailed neuromuscular model of human walking. They showed that, with one cable-driven biarticular actuator, the exoskeleton can reduce subject muscle activation and metabolic cost, while ensuring balance stability during walking.

A group of three articles focused on experimental and theoretical methods for the evaluation of human-like performance of bipedal machines, in either real or simulated environments. Stasse et al. investigated the effect of temperatures variation on the walking performance of the HRP-2 robot, computing a set performance indicators previously defined by Torricelli et al. (2015) across different tasks, such as standing on tilting and horizontally moving surfaces, and walking during pushes, uneven or inclined surfaces, with the aim of testing the capability of a humanoid robot in real-life situations. Okajima et al. developed a control algorithm able to generate different human-like bipedal behaviors by adjusting a few signals in a symbolized control space, with the help of a tacit learning approach. This work has potential benefit not only in simplifying the control of a humanoid robot, but also in understanding the mechanisms at the basis of human-like walking and standing. Iosa et al. propose the golden ratio as the trade-off between advancement and equilibrium managed during walking, providing additional theoretical evidence in favor of the golden ratio as a descriptor of harmonic gait. This approach may be extended to assess the harmony in humanoid robotic walkers or neurorobots for rehabilitation.

## Conclusions and Future Perspectives

The large number of articles collected in this Research Topic are in itself proof of the substantial interest that different research communities have in the definition of reliable metrics of bipedal performance.

In the prosthetic field, we observed a clear need of metrics able to predict the interaction between the prosthetic device and human, as well as to measure the functional effects of walking in a real environment, in presence of transitions between different terrains. In the exoskeleton field, there is an increasing interest in identifying strategies for more adjustable/adaptable machines, under the perspective of control, actuation, or ergonomics. In the humanoids field, a promising trend is to test the performance in outdoor-like scenarios under a wide variety of perturbations, e.g., moving grounds or pushes.

The high heterogeneity of the approaches even further highlights the need for a unified framework in which these views are brought together and can be easily shared across the research community. Promising efforts in this direction are currently promoted by two European initiatives: the EUROBENCH project “European Robotic Framework for Bipedal Locomotion Benchmarking” (Torricelli and Pons, 2019, www.eurobench2020.eu) and the COST action CA16116 “Wearable Robots for Augmentation, Assistance or Substitution of Human Motor Functions” (https://www.cost.eu/actions/CA16116). Both initiatives aim to establish common methodologies and promote the discussion on standardized assessment of robotic systems, focused but not limited to bipedal locomotion.

This research field needs to address some important bottlenecks. First, the great majority of methods proposed in the literature are applied to individual devices or specific experimental settings. While many works claim a potential applicability of their metrics across different laboratory conditions or devices, very few of them actually have demonstrated such wider applicability. Nevertheless, this issue is critical for benchmarking, of which the principal goal is to compare different systems under reproducible conditions. This is particularly relevant considering the important trade-off between simplicity and reproducibility, validity of a benchmark and relevance of the outcome, considering the many subtle differences in applications, e.g., differences across industrial environments, or differences in pathologies across subjects.

We also observed an intrinsic difficulty in comparing bipedal robot performance with human performance. The concept of “human likeness,” intuitively easy to grasp, becomes hard to define under a rigorous and systematic way, especially in robotics systems that have intrinsic differences (e.g., kinematic configuration) with humans, or when the human is not the golden standard for performance (similarly to the case of chess players). New challenges and questions (e.g., is it useful to compare robots with humans?) may be object of future research.

We believe that reaching an international consensus on these topics will be extremely beneficial to boost the process of finding reliable methods to test and compare different systems and identifying robust metrics to measure the Technology Readiness Level (TRL) of new robotic solutions.

## Author Contributions

DT drafted the initial version of the manuscript. All authors have made substantial and iterative contribution to the work, and approved its final version for publication.

### Conflict of Interest

The authors declare that the research was conducted in the absence of any commercial or financial relationships that could be construed as a potential conflict of interest.
